# Predicting Laparotomy Outcomes Using the American College of Surgeons National Surgical Quality Improvement Program (ACS-NSQIP) and National Emergency Laparotomy Audit (NELA): A Retrospective Analysis From Two Centres

**DOI:** 10.7759/cureus.106391

**Published:** 2026-04-03

**Authors:** Malcolm Irem, Jessica Wang Yean Ping, Georgia Tooth, Yaseen Othman, Mohammad Badr Almoshantaf, Panagiotis Drymousis, Asantha Jayaweera, Hemant Sheth

**Affiliations:** 1 General Surgery, London North West University Healthcare NHS Trust, London, GBR; 2 Anaesthesia, London North West University Healthcare NHS Trust, London, GBR

**Keywords:** emergency surgery, frailty, laparotomy, mortality, peri-operative outcomes, post-operative care, pre-operative assessment

## Abstract

Background

Risk prediction in emergency laparotomy supports peri-operative decision-making, counselling and resource allocation in acutely unwell surgical patients. In the United Kingdom (UK) practice, the National Emergency Laparotomy Audit (NELA) score is widely used because it is specific to emergency laparotomy, whereas the American College of Surgeons National Surgical Quality Improvement Program (ACS-NSQIP) calculator incorporates broader comorbidity and functional status variables. Direct comparisons between these tools in UK emergency laparotomy cohorts remain limited. The primary objective of this study was to compare the discriminatory performance of pre-operative NELA, post-operative NELA and ACS-NSQIP for predicting 30-day mortality after emergency laparotomy. Secondary objectives were to compare overall prediction accuracy using the Brier score and to describe threshold-based test characteristics.

Methods

We performed a retrospective observational two-centre cohort study at two district general hospitals. Adult patients undergoing emergency laparotomy between November 2023 and October 2024 were identified from electronic records. Mortality risk was calculated using ACS-NSQIP, pre-operative NELA and post-operative NELA. Pre-operative NELA was derived from variables available at the time of operative decision-making, whereas post-operative NELA incorporated intra-operative variables. The primary outcome was 30-day post-operative mortality. Model performance was evaluated using the area under the receiver operating characteristic curve (AUC) and Brier score. DeLong's test was used to compare AUCs.

Results

A total of 138 patients were included; 30-day mortality was 11.6% (16/138). Post-operative NELA had the highest AUC at 0.878 (95% CI: 0.762-0.943), followed by pre-operative NELA at 0.863 (95% CI: 0.762-0.943) and ACS-NSQIP at 0.826 (95% CI: 0.765-0.942). Brier scores were 0.077, 0.078 and 0.094, respectively. Although post-operative NELA showed numerically higher discrimination and lower overall prediction error, differences between models were not statistically significant, including comparison with ACS-NSQIP (DeLong's test; p = 0.616). At a descriptive 10% mortality threshold, post-operative NELA had higher sensitivity than ACS-NSQIP but slightly lower specificity.

Conclusion

In this two-centre retrospective cohort, all three models demonstrated good performance for predicting 30-day mortality after emergency laparotomy. Post-operative NELA performed numerically best, but differences between models were statistically non-significant and should be interpreted cautiously given the small sample size and limited number of mortality events. These findings are exploratory and support further evaluation in larger multicentre cohorts.

## Introduction

Emergency laparotomy remains one of the highest-risk procedures performed in contemporary surgical practice, with short-term mortality markedly exceeding that seen in elective surgery [1,2]. Patients often present with sepsis, bowel ischaemia, perforation, physiological instability and multiple comorbidities, leaving limited time for optimisation before intervention. In this setting, accurate peri-operative risk estimation is clinically important: it can support shared decision-making, senior review, post-operative destination planning and prioritisation of critical care resources.

Two commonly used tools for this purpose are the National Emergency Laparotomy Audit (NELA) risk model and the American College of Surgeons National Surgical Quality Improvement Program (ACS-NSQIP) surgical risk calculator. NELA was developed specifically for adult emergency laparotomy patients using United Kingdom (UK) data and predicts 30-day mortality in this population [3]. Its appeal lies in its procedure-specific design and relative practicality within acute surgical pathways. By contrast, ACS-NSQIP was developed as a broader surgical risk calculator and incorporates a wider range of patient-level variables, including comorbidity burden, American Society of Anesthesiologists (ASA) grade and functional status [4]. Although not specific to emergency laparotomy, this broader case-mix modelling may be advantageous in selected patients with complex medical backgrounds.

Previous studies have shown that NELA performs well compared with older risk tools such as the Portsmouth Physiological and Operative Severity Score for the enumeration of Mortality and Morbidity (P-POSSUM) in emergency general surgery [5]. Comparisons between NELA and ACS-NSQIP have also been reported internationally [6], but direct evaluation in UK emergency laparotomy cohorts remains relatively limited. This matters because the two models differ not only in the variables they include but also in their intended context of use: NELA is tightly aligned to emergency laparotomy, whereas ACS-NSQIP offers a broader peri-operative framework. Understanding how these tools perform relative to one another in routine UK practice may help clinicians interpret risk estimates more appropriately rather than assuming that one model is universally preferable.

The primary objective of this study was to compare the discriminatory performance of pre-operative NELA, post-operative NELA and ACS-NSQIP for predicting 30-day mortality following emergency laparotomy. Secondary objectives were to compare overall prediction accuracy using the Brier score and to describe model performance at a pragmatic 10% mortality threshold, as well as mortality estimates within clinically relevant subgroups. We framed this study as an exploratory retrospective comparison/local validation study rather than a definitive superiority analysis.

## Materials and methods

Study design and setting

This was a retrospective observational two-centre cohort study conducted at two district general hospitals, namely, Ealing Hospital and Northwick Park Hospital, based in London, UK (both fall under London North West University Healthcare NHS Trust). The study reviewed adult patients who underwent emergency laparotomy between November 2023 and October 2024.

This study was conducted as a retrospective service evaluation using anonymised routinely collected clinical data. No patient contact or intervention was involved, and the project did not influence clinical management. In accordance with the NHS Health Research Authority guidance, formal research ethics committee approval was not required. All patient information was anonymised prior to analysis.

Participants

Eligible patients were aged 18 years or older and underwent emergency laparotomy during the study period. Exclusion criteria were age under 18 years, elective surgery and insufficient clinical information to calculate both ACS-NSQIP and NELA risk estimates. No imputation of missing values was performed; cases missing essential variables for either model were excluded from the final analytic cohort.

Included operations comprised a range of emergency abdominal procedures, including hemicolectomy, Hartmann's procedure, adhesionolysis, appendicectomy and cholecystectomy. Appendicectomy and cholecystectomy were included because the study aimed to reflect the spectrum of unplanned open abdominal operations encountered in local emergency surgical practice. However, we recognise that inclusion of lower-risk procedures within an "emergency laparotomy" cohort is not universal across studies and may influence overall mortality and model calibration; this is acknowledged as a limitation.

Data collection

Clinical data was extracted retrospectively from electronic health records using a standardised proforma. Data extraction was undertaken independently by multiple authors. A subset of cases was cross-checked by a second reviewer, with discrepancies resolved by consensus. Formal inter-rater reliability testing was not performed.

For pre-operative NELA, variables were reconstructed from documentation available before surgery, including physiological observations, comorbidity data and pre-operative investigations recorded at the time of operative decision-making [7]. Post-operative NELA was then calculated using the same platform after incorporating intra-operative variables such as operative severity, contamination, blood loss and operative findings [7]. All variables were entered using standard NELA definitions.

For ACS-NSQIP, the publicly available risk calculator was used [8]. A single principal current procedural terminology (CPT) code was selected for each patient, even where more than one procedure code might have been applicable. The "alternative treatment options" field was set to "none" for standardisation, and surgeon-adjusted risk was set to "1 (no adjustment necessary)" for all patients. These decisions were made to ensure consistency of retrospective application across the cohort. However, we recognise that this approach may not fully capture procedural complexity or clinician judgement and may have affected ACS-NSQIP predictive accuracy.

Outcomes

The primary outcome was 30-day post-operative mortality. Secondary descriptive outcomes included subgroup mortality by ASA class and age and threshold-based sensitivity/specificity at a 10% predicted mortality cut-off. The 10% threshold was chosen as a pragmatic descriptive threshold for clinical interpretability rather than as a pre-specified superiority threshold.

Statistical analysis

Model discrimination was assessed using the area under the receiver operating characteristic curve (AUC), with 95% confidence intervals. DeLong's test was used to compare AUCs between models. Overall prediction accuracy/calibration was assessed using the Brier score, calculated as the mean squared difference between predicted probability and observed 30-day mortality outcome.

Data management was performed in Microsoft Excel (Microsoft Corp., Redmond, WA, USA), and statistical analyses including receiver operating characteristic (ROC) curve/AUC estimation and DeLong testing were performed using Python (version 3.11.8) (Python Software Foundation, Wilmington, DE, USA). Brier score calculations were performed using the standard formula applied to individual predicted probabilities and binary mortality outcomes. Calibration slope, calibration intercept and calibration plots across risk deciles were not available in the present analysis and are therefore acknowledged as limitations.

Because this was a pragmatic retrospective cohort including all eligible cases during the defined study period, no a priori sample size or power calculation was undertaken. Given the limited number of mortality events, the study may have been underpowered to detect small but clinically meaningful differences between models, increasing the risk of type II error.

Hospital length of stay (LOS) was summarised descriptively using the median and range because of its skewed distribution. Extreme LOS values, including prolonged admissions, were retained where verified as true clinical observations rather than data-entry errors.

## Results

Patient characteristics

As shown in Table 1, a total of 138 patients underwent emergency laparotomy at both district general hospitals between November 2023 and October 2024. The cohort represented a diverse range of adult surgical patients, with a median age of 66 years (range 19-96), highlighting the inclusion of both younger and elderly patients undergoing high-risk emergency abdominal surgery. The gender distribution was slightly skewed toward males, with 72 male patients (52.2%) and 66 female patients (47.8%). 

**Table 1:** Patient demographics LOS: length of stay; ASA: American Society of Anesthesiologists Physical Status Classification System

Parameter		n
Number of patients		138
Age (years) (median)		66 (19-96)
Sex	Male	72 (52.2%)
	Female	66 (47.8%)
ASA score	I	4 (2.9%)
	II	49 (35.5%)
	III	59 (42.8%)
	IV	25 (18.1%)
	V	1 (0.7%)
LOS (days) (median)		24.5 (0-238)

The physiological status of patients was assessed using the ASA Physical Status Classification System, a widely recognised pre-operative risk stratification tool. Four patients were classified as ASA I (healthy). Most patients were either ASA II (n = 49; 35.5%), indicating mild systemic disease, or ASA III (n = 59; 42.8%), reflecting patients with severe systemic disease. Notably, 25 patients (18.1%) were categorised as ASA IV, suggesting severe systemic disease posing a constant threat to life, and one patient (0.7%) was classed as ASA V, indicating a morbid patient not expecting to survive for 24 hours, with or without surgery. 

The median hospital LOS for the cohort was 24.5 days, with a wide range from 0 to 238 days, indicating significant variation in post-operative recovery and care needs. This variability is likely reflective of differences in patient comorbidities, intra-operative findings, post-operative complications and access to intensive care or specialist support. 

Overall, the demographic and clinical profile of this cohort represents a typical high-risk emergency surgical population, providing a robust sample for evaluating the predictive accuracy of pre-operative and post-operative risk assessment tools. 

Mortality

Thirty-day post-operative mortality was 11.594% (16/138). Fifteen deaths occurred before discharge, whereas one occurred within 30 days after discharge. Mortality was concentrated in higher-risk subgroups: among inpatient deaths, seven patients were ASA IV and eight were ASA III. Within the cohort, 15 of 16 deaths occurred in patients aged 65 years or older, consistent with the established relationship between older age and worse emergency surgical outcomes [2,9].

The average predicted mortality by model, ASA class and age group is shown in Table 2. In ASA IV patients (n = 25), mean predicted mortality estimates were 21.488% for pre-operative NELA, 21.326% for post-operative NELA and 19.846% for ACS-NSQIP, compared with an observed mortality of 28.000%. These subgroup findings are descriptive only and should be interpreted cautiously because of the small numbers involved.

**Table 2:** Average mortality predictions and observed mortality arranged by ASA class and age All values are rounded to three decimal places. ASA: American Society of Anesthesiologists Physical Status Classification System; NELA: National Emergency Laparotomy Audit; ACS-NSQIP: American College of Surgeons National Surgical Quality Improvement Program

ASA class or age	Number of patients	Average pre-op NELA (%)	Average post-op NELA (%)	Average ACS-NSQIP (%)	Observed mortality on discharge	Observed mortality (%)	Observed mortality within 30 days of discharge	Observed mortality within 30 days of discharge (%)	Total mortality	Total mortality (%)
All	138	9.648	10.037	8.356	15	10.870	1	0.725	16	11.594
ASA I	4	0.283	0.495	0.800	0	0.000	0	0.000	0	0.000
ASA II	49	2.123	2.198	1.704	0	0.000	1	2.041	1	2.041
ASA III	59	10.699	11.603	8.249	8	13.559	0	0.000	8	13.559
ASA IV	25	21.488	21.326	19.846	7	28.000	0	0.000	7	28.000
ASA V	1	29.890	29.890	52.000	0	0.000	0	0.000	0	0.000
Age <65	64	3.994	3.959	4.278	1	1.563	0	0.000	1	1.563
Age >65	74	14.151	14.887	11.495	14	18.919	1	1.351	15	20.270

ROC curve analysis: discriminatory ability

**Figure 1:**
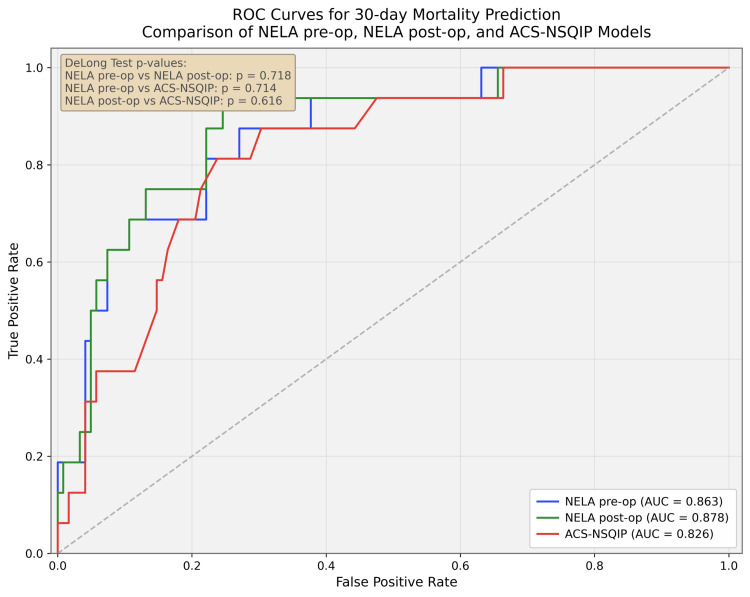
ROC curve comparing the prognostic ability of mortality for pre-operative NELA and mortality for ACS-NSQIP scores in predicting 30-day mortality ROC: receiver operating characteristic; NELA: National Emergency Laparotomy Audit; ACS-NSQIP: American College of Surgeons National Surgical Quality Improvement Program

All three models demonstrated good discrimination for 30-day mortality. Post-operative NELA had the highest AUC at 0.878 (95% CI: 0.762-0.943), followed by pre-operative NELA at 0.863 (95% CI: 0.762-0.943) and ACS-NSQIP at 0.826 (95% CI: 0.765-0.942) (Figure 1). Although post-operative NELA had the numerically highest AUC, confidence intervals overlapped substantially.

DeLong testing did not demonstrate statistically significant differences between models. In particular, the comparison between post-operative NELA and ACS-NSQIP was non-significant (p = 0.616). These findings suggest comparable discriminatory performance within the limits of this sample.

Brier score: calibration

Brier scores were lowest for post-operative NELA (0.0769), followed closely by pre-operative NELA (0.0782), with ACS-NSQIP showing a higher score (0.0940). This indicates that all three models performed reasonably well overall, with numerically lower prediction error for the NELA models. However, because calibration slope, intercept and graphical calibration assessment were not performed, conclusions regarding calibration should be considered limited.

At a descriptive 10% mortality threshold, post-operative NELA showed higher sensitivity than ACS-NSQIP (0.938 vs 0.813) but slightly lower specificity (0.730 vs 0.762). These threshold-based findings were exploratory and were not used to support claims of superiority.

Interpretation

All three models demonstrated good overall performance in predicting 30-day mortality, with high AUC values and low Brier scores indicating acceptable discrimination and calibration. The post-operative NELA model showed the lowest Brier score and the highest AUC (0.878). However, differences between the post-operative NELA model and both the pre-operative NELA and ACS-NSQIP models were not statistically significant (DeLong's test; p = 0.718 and p = 0.616, respectively).

These findings indicate that the predictive performance of the three models was comparable within this cohort. While small differences in discrimination and calibration metrics were observed, their clinical significance is uncertain. Each model may therefore have potential utility for risk estimation in patients undergoing emergency laparotomy, with differences primarily relating to model structure and timing of variable availability.

## Discussion

In this retrospective two-centre cohort of 138 adult patients undergoing emergency laparotomy, all three models evaluated (pre-operative NELA, post-operative NELA and ACS-NSQIP) demonstrated good discrimination for 30-day mortality, with AUC values ranging from 0.826 to 0.878. Post-operative NELA showed the numerically highest AUC and lowest Brier score, but the observed differences were small, confidence intervals overlapped substantially and formal comparison by DeLong's test did not show statistical significance. Accordingly, these findings suggest that the predictive performance of ACS-NSQIP and both NELA models was broadly comparable within this dataset and the results do not demonstrate a clear statistical superiority of any single model.

This distinction is important. The original intention of the study was to explore how three commonly used risk tools perform in a real-world UK emergency laparotomy cohort. Given the small sample size and only 16 mortality events, the study was underpowered for definitive comparative inference. This raises the possibility of type II error, meaning clinically relevant differences may not have been detected. The findings should therefore be viewed as exploratory and hypothesis-generating rather than confirmatory.

The study remains clinically relevant because the models compared differ meaningfully in purpose and structure. NELA was designed specifically for emergency laparotomy using UK data [3], which likely explains its strong face validity and practical appeal in acute care pathways. ACS-NSQIP, in contrast, is a broader surgical calculator incorporating additional patient-level information such as comorbidity burden and functional status [4]. In practice, this may make ACS-NSQIP attractive when more detailed pre-operative information is available, whereas NELA may be more practical in time-pressured emergency settings. A cautious practical interpretation is that pre-operative NELA and ACS-NSQIP may both support pre-operative counselling and escalation discussions, while post-operative NELA may be helpful for post-operative risk stratification once intra-operative findings are known. However, this study was not powered to support policy-level recommendations regarding preferred routine use.

The overall 30-day mortality of 11.6% is consistent with published outcomes for emergency abdominal surgery in similar populations [1,6]. As expected, mortality was concentrated among older and higher-ASA patients, in keeping with previous literature linking age, frailty and physiological reserve to poor post-operative outcomes [2,9]. The descriptive ASA IV subgroup findings suggested that both NELA models produced mortality estimates closer to observed mortality than ACS-NSQIP, but the subgroup sample was small, and these findings should not be overinterpreted.

Several methodological issues require emphasis. First, appendicectomy and cholecystectomy were included within the emergency laparotomy cohort to reflect local real-world practice, but these are lower-risk procedures in many contexts, and their inclusion may have reduced overall mortality and influenced model calibration. Because of the limited event count, we did not perform a formal higher-risk-only subgroup analysis; such analyses would be more appropriately undertaken in a larger dataset.

Second, both pre-operative NELA and ACS-NSQIP scores were derived retrospectively from existing documentation. Although pre-operative NELA was reconstructed using variables recorded at the time of operative decision-making, retrospective data abstraction may still introduce misclassification or hindsight bias. Third, the ACS-NSQIP methodology required standardised assumptions for retrospective scoring. We selected a single principal CPT code, set alternative treatment options to "none," and did not apply surgeon adjustment [7]. This improved consistency across the cohort but may have reduced the ability of ACS-NSQIP to reflect the full complexity of emergency cases, particularly where multiple concurrent procedures or strong surgeon-level contextual judgement would have altered risk estimates.

A further limitation is the restricted calibration assessment. We used the Brier score to quantify overall prediction error, but did not report calibration intercepts, slopes or observed-versus-predicted plots across risk deciles. Although the Brier score provides useful summary information, it does not fully characterise calibration performance, and future work should include more comprehensive calibration analyses. Additional important sources of heterogeneity, including operative indication, degree of peritoneal contamination, sepsis, frailty and procedure severity, were not modelled separately in this study and may have influenced both observed outcomes and apparent model performance.

Despite these limitations, the study has strengths. It addresses a clinically important question using accepted prediction-model performance metrics, includes a two-centre real-world cohort and directly compares a UK emergency laparotomy-specific tool with a widely used broader surgical calculator. These findings add local validation data to an area where UK-specific head-to-head comparisons remain relatively limited.

Future studies should include larger multicentre cohorts with sufficient event numbers to support more robust comparative analyses, formal calibration assessment and stratified evaluation by procedure risk, frailty and pathology. Such work would be better placed to determine whether one model is meaningfully preferable in specific emergency surgical subgroups.

## Conclusions

In this retrospective two-centre cohort, pre-operative NELA, post-operative NELA and ACS-NSQIP all showed good performance for predicting 30-day mortality after emergency laparotomy. Post-operative NELA had the numerically highest AUC and lowest Brier score, but differences between models were statistically non-significant and confidence intervals overlapped substantially. The findings therefore support broadly comparable performance rather than definitive superiority of any one model.

From a practical perspective, model choice may depend on the stage of care and the information available. Pre-operative NELA and ACS-NSQIP may both assist pre-operative counselling and escalation discussions, whereas post-operative NELA may be useful once operative findings are known. However, given the retrospective design, heterogeneous case mix, limited calibration analysis and small number of mortality events, these results should be interpreted as exploratory local validation data. Larger multicentre studies are required before stronger comparative or policy recommendations can be made.

## References

[1] Pearse RM, Moreno RP, Bauer P (2012). Mortality after surgery in Europe: a 7 day cohort study. Lancet.

[2] Hajibandeh S, Hajibandeh S, Shah J (2021). The risk and predictors of mortality in octogenarians undergoing emergency laparotomy: a multicentre retrospective cohort study. Langenbecks Arch Surg.

[3] Eugene N, Oliver CM, Bassett MG (2018). Development and internal validation of a novel risk adjustment model for adult patients undergoing emergency laparotomy surgery: the National Emergency Laparotomy Audit risk model. Br J Anaesth.

[4] Bilimoria KY, Liu Y, Paruch JL, Zhou L, Kmiecik TE, Ko CY, Cohen ME (2013). Development and evaluation of the universal ACS NSQIP surgical risk calculator: a decision aid and informed consent tool for patients and surgeons. J Am Coll Surg.

[5] Thahir A, Pinto-Lopes R, Madenlidou S, Daby L, Halahakoon C (2021). Mortality risk scoring in emergency general surgery: are we using the best tool?. J Perioper Pract.

[6] (2020). High-risk emergency laparotomy in Australia: comparing NELA, P-POSSUM, and ACS-NSQIP calculators. J Surg Res.

[7] (2025). Parsimonious NELA risk calculator. https://data.nela.org.uk/riskcalculator/.

[8] (2025). ACS NSQIP surgical risk calculator. https://riskcalculator.facs.org/RiskCalculator/index.jsp.

[9] Snitkjær C, Rehné Jensen L, Í Soylu L (2024). Impact of clinical frailty on surgical and non-surgical complications after major emergency abdominal surgery. BJS Open.

